# Estimating the Prevalence of Autism Spectrum Disorder in New South Wales, Australia: A Data Linkage Study of Three Routinely Collected Datasets

**DOI:** 10.1007/s10803-022-05887-3

**Published:** 2023-01-18

**Authors:** Timothy C. Nielsen, Natasha Nassar, Kelsie A. Boulton, Adam J. Guastella, Samantha J. Lain

**Affiliations:** 1 Children’s Hospital Westmead Clinical School, Faculty of Medicine and Health, University of Sydney, Camperdown, NSW 2006 Australia; 2 Brain and Mind Centre, Children’s Hospital Westmead Clinical School, Faculty of Medicine and Health, University of Sydney, Camperdown, NSW Australia

**Keywords:** Autism, Prevalence, Data linkage, Australia

## Abstract

Routinely collected data help estimate the prevalence of autism spectrum disorder (ASD) in jurisdictions without active autism surveillance. We created a population-based cohort of 1,211,834 children born in 2002–2015 in New South Wales, Australia using data linkage. Children with ASD were identified in three datasets – disability services, hospital admissions, and ambulatory mental health data. The prevalence of ASD in the cohort was 1.3% by age 12 and prevalence at age 6 increased an average of 4.1% per year (95% Confidence Interval, 3.3%, 4.8%). Most children with ASD were identified in disability services data (87%), although data linkage identified 1,711 additional cases that were more likely female, older at first contact, and living in major cities and less disadvantaged areas.

**Supplementary Information:**

The online version contains supplementary material available at 10.1007/s10803-022-05887-3.

Autism spectrum disorder (ASD) is a neurodevelopmental disorder characterised by deficits in communication and social interaction and repetitive patterns of behaviour, interests or activities that appears in the early developmental period (*DSM-5*, 2013). ASD is a leading cause of disability for children under 5 years of age and is two to five times more common among male children (Baxter et al., 2015; Lord et al., 2020). ASD is highly heritable, but its aetiology is complex, with environmental and epigenetic effects thought to play a part (Lord et al., 2020). The reported prevalence of ASD has increased over time, from less than 0.05% in studies the 1970s (Elsabbagh et al., 2012) to 0.8% in the 2010 Global Burden of disease study (Baxter et al., 2015) and 2.3% in 2018 from an active autism surveillance program in the United States (Maenner et al., 2021). There are many potential causes of this increase, including increased awareness among parents and providers, inclusion of milder cases with expanded diagnostic criteria, or an increase in true prevalence (Elsabbagh et al., 2012; Lyall et al., 2017; Zablotsky et al., 2019).

There are substantial differences in estimates from contemporaneous studies. For example, consider three studies estimating the national prevalence of ASD in children in the United States in 2016/2017: The first reported an ASD prevalence of 1.2% of school-aged children based on special education data (Safer-Lichtenstein et al., 2021), the second reported a prevalence of 1.9% of 8 year old children based on abstraction of medical, education, and service records (Maenner et al., 2020), and the third reported a prevalence of 2.5% of children aged 3 to 17 based on a survey of parents (Zablotsky et al., 2019). Similarly, a study comparing autism registries in Denmark, Finland, France, and Iceland reported an array of ASD prevalence at age 8 years from 0.5% in Southeast France to 3.1% in Iceland (Delobel-Ayoub et al., 2020). Some of this variability may be related to true differences between populations, but differences in data sources and data collection methods play an important role.

Accurately estimating the prevalence of ASD in specific populations is crucial for planning and providing services. Active surveillance systems that seek out potential ASD cases via abstraction and review of medical, special education, and support service records are arguably the gold standard for estimating prevalence. Many jurisdictions do not have the resources to implement active surveillance systems, however routinely collected data can offer an important alternative for identifying children with ASD at the population level. Furthermore, data linkage methods, which identify individuals in multiple data sources and combine their information, can improve case identification compared to individual data sources.

New South Wales (NSW), Australia’s most populous state, currently lacks an active surveillance system or centralized registry for ASD. However, children diagnosed with ASD can be identified in routinely collected data when they access healthcare and other support services. This currently includes publicly funded disability services, such as early intervention services, as well as hospital admissions and ambulatory mental health visits. Records of these contacts and relevant diagnoses are documented in databases maintained by the NSW Ministry of Health. The aims of this study were to use data linkage to (1) estimate and examine trends in the prevalence of ASD in NSW, Australia and (2) compare the characteristics of individuals identified in these three routinely collected data sources to explore the impact of data linkage. We predict these data sources will identify distinct populations and that data linkage will improve ASD case identification compared to individual sources.

## Methods

### Study Population

The study included all children born in NSW between July 1, 2002 and June 30, 2015 identified from the NSW Perinatal Data Collection, a database of all births ≥ 20 weeks gestation or ≥ 400 g birthweight comprising information on maternal socio-demographics, pregnancy and birth outcomes, and infant characteristics. Children in the study cohort were followed up from birth until December 31, 2017 via linkage to three population-based datasets to identify ASD diagnoses: (i) children’s disability services records, (ii) hospital admissions, and (iii) ambulatory mental health encounters. Probabilistic linkage of the data sets was conducted by the NSW Centre for Health Record Linkage.

### Disability Services Records

Disability services data were obtained from the NSW Family and Community Services Disability Dataset beginning on July 1, 2003. During the study period, the Australian government funded services for people with disabilities under the National Disability Agreement, requiring all service providers receiving public funding to regularly report information outlined in the Disability Services National Minimum Dataset (Australian Institute of Health and Welfare, 2016). This information includes the individual’s first date of service and subsequent services and their relevant primary and other significant disability type(s) selected from 12 disability groups (Supplementary Table S1).

To be eligible to receive disability services for autism, individuals must have received a relevant diagnosis from a specialist multi-disciplinary team, pediatrician, psychiatrist, or clinical psychologist experienced in the assessment of pervasive developmental disorders and assessed using the current Diagnostic and Statistical Manual of Mental Disorders (DSM-IV during the study period). (Florio & Trollor, 2015; Reppermund et al., 2017). Receipt of services is based on an individual’s needs rather than their ability to pay (Australian Government Department of Social Services, 2011).

### Hospital Admission Data

Hospital admission data were obtained from the NSW Admitted Patient Data Collection. This is a state-wide dataset of all public and private hospital admissions. Admission records include a primary and up to 50 additional related diagnoses coded according to the 10th revision of the International Classification of Diseases Australian modification (ICD10-AM).

### Ambulatory Mental Health Data

Ambulatory mental health data were obtained from the NSW Mental Health Ambulatory Data Collection. This dataset includes all public mental health services for non-admitted patients, including day programs, psychiatric outpatient visits, and outreach services. These data do not include general practitioner and private specialist encounters or routine health assessments. One mental health diagnosis related to each service contact is recorded using ICD10-AM coding.

### Study Outcomes

The primary outcome of the study was a recorded diagnosis of ASD specified in one or more of the data sources. For disability services data, ASD diagnoses were identified by classification in the “autism” disability group, which requires prior diagnosis with autism, Asperger’s syndrome or other pervasive developmental disorder based on DSM-IV criteria. For hospital admission and ambulatory mental health data, ASD diagnoses were identified by one or more encounter with a recorded diagnosis of ICD10-AM code F84. Detailed diagnostic information was not available in the three datasets and disability services data does not disaggregate specific pervasive developmental disorders. For the purposes of this study, the term ASD was chosen to describe this outcome.

In addition, intellectual disability (ID) was included as a secondary outcome to examine whether this was offset by ASD during the study period. This was identified in the disability services data by classification in the “intellectual” disability group. For all outcomes, age at first contact (service, admission, or encounter) was ascertained and if a child had an outcome recorded in multiple datasets, the earliest relevant date was used for age at first contact. Given the data sources, a child’s age at first contact is not equivalent to age at diagnosis.

### Data Analysis

The number of children with ASD were identified in each data source, and characteristics of children including child sex, age at first contact, socioeconomic status, and remoteness of residence at time of birth were compared using Pearson’s chi-squared tests. Socioeconomic status (quintiles) and remoteness (major city, inner regional, outer regional, remote area) were coded using the Socio-Economic Indexes for Areas (SEIFA) and the Accessibility and Remoteness Index of Australia (ARIA) from the Australian Bureau of Statistics (Australian Bureau of Statistics, 2013 A; Australian Bureau of Statistics, 2013B). The proportion of children identified in each and multiple data sources was determined.

Prevalence of ASD by child sex was estimated at four age points (3 years, 6 years, 9 years, and 12 years old) for each data source and compared. All children in the cohort that reached a given age (3, 6, 9 or 12 years) by the end of the follow-up period contributed to the age-specific denominator. Estimates were calculated for the entire study period and by individual financial years of birth. Potential trends in prevalence at age 6 years by year of birth were examined by calculating average annual percent change using negative binomial regression to control for overdispersion. An age of 6 years was selected to balance the optimal proportion of cases identified and the years of available data decreasing with age.

In addition, the proportion of children identified in disability services data with an ID diagnosis was calculated by year of birth, and trends in ID prevalence at age 6 years were examined and compared to trends in ASD. The prevalence of ID was selected as a comparison because it was expected to remain stable over the study period compared to the prevalence of ASD which was expected to increase (Nevison & Blaxill, 2017). All analyses were performed using SAS 9.4 software (SAS Institute, Cary, North Carolina, USA). Ethics approval for the study was obtained from the NSW Population and Health Services Research Ethics Committee.

## Results

A total of 1,211,834 children were born in NSW from 1 July, 2002 to 30 June, 2015 and included in the study cohort. Overall, 12,921 children (1.1%) had a recorded diagnosis of ASD during the follow up period; of those children, 86.8% were identified in disability services data, 22.5% in hospital admission data, and 7.5% in ambulatory mental health data, with some overlap between sources (Table 1).

**Table 1:** Characteristics of children identified with autism spectrum disorder across three routinely collected data sources in New South Wales, Australia, born 2002–2015

Characteristics	Total Population	Autism Spectrum Disorder				p value^a^	p value^b^
		Any Source	Disability Services	Hospital Admissions	Ambulatory Mental Health		
	(n=1,211,834)	(n=12,921)	(n=11,210)	(n=2,913)	(n=972)		
	n (%)	n (%)	n (%)	n (%)	n (%)		
Child sex						<0.001	<0.001
Male	622,949 (51.4)	10,160 (78.6)	8879 (79.2)	2270 (77.9)	760 (78.2)		
Female	588,374 (48.6)	2756 (21.3)	2327 (20.8)	642 (22.0)	211 (21.7)		
Age at first contact (years)							<0.001
0–2		3709 (28.7)	3545 (31.6)	288 (9.9)	15 (1.5)		
3–5		6526 (50.5)	5988 (53.4)	1248 (42.8)	148 (15.2)		
6–8		1611 (12.5)	1085 (9.7)	815 (28.0)	311 (32.0)		
9–11		771 (6.0)	467 (4.2)	385 (13.2)	321 (33.0)		
12–15		304 (2.4)	125 (1.1)	177 (6.1)	177 (18.2)		
Residence at birth						<0.001	<0.001
Major city	936,467 (77.3)	9168 (71.0)	7849 (70.0)	2213 (76.0)	733 (75.4)		
Inner regional	199,360 (16.5)	2988 (23.1)	2701 (24.1)	546 (18.7)	159 (16.4)		
Outer regional	54,080 (4.5)	662 (5.1)	569 (5.1)	134 (4.6)	66 (6.8)		
Remote/Very remote	7067 (0.6)	60 (0.5)	52 (0.5)	13 (0.5)	12 (1.2)		
Missing	14,860 (1.2)	43 (0.3)	39 (0.4)	7 (0.2)	2 (0.2)		
Socioeconomic disadvantage at birth						<0.001	<0.001
Quintile 1 – Most disadvantaged	264,570 (21.8)	3258 (25.2)	2826 (25.2)	759 (26.1)	259 (26.7)		
Quintile 2	285,574 (23.6)	3794 (29.4)	3338 (29.8)	796 (27.3)	257 (26.4)		
Quintile 3	230,767 (19.0)	2547 (19.7)	2247 (20.0)	579 (19.9)	125 (12.9)		
Quintile 4	186,186 (15.4)	1749 (13.5)	1475 (13.2)	417 (14.3)	168 (17.3)		
Quintile 5 – Least disadvantaged	229,877 (19.0)	1530 (11.8)	1285 (11.5)	355 (12.2)	161 (16.6)		
Missing	14,860 (1.2)	43 (0.3)	39 (0.4)	7 (0.2)	2 (0.2)		

Categories are not mutually exclusive. ^a^Pearson’s Chi Squared test comparing characteristics of children with and without an ASD diagnosis (from any source)

^b^Pearson’s Chi-Squared test comparing characteristics of children with an ASD diagnosis identified in different data sources (using mutually exclusive groups).

Compared to all children born in NSW, children identified with ASD were more likely to be male (78.6% vs. 51.4%) with a similar 3:1 male-to-female sex distribution in each data source. Based on residence at birth, ASD cases were more commonly identified in inner regional areas (outside of but closest to major cities) and more disadvantaged areas. Distributions were similar between sources, although disability services data identified more children in inner regional areas and ambulatory mental health data identified more children in less disadvantaged areas compared to other sources. Children identified with ASD in disability services data were younger at first contact (median 3 years, Interquartile range (IQR) 2–4) compared to hospital admissions (median 5 years, IQR 4–8) and ambulatory mental health data (median 9 years, IQR 6–11) (Table 1).

Most cases of ASD (72%) were identified by disability services data alone and only a small proportion of cases (16%) were identified in multiple sources (Supplementary Table S2). Overall, 86.8% of children with ASD were identified in disability services data. Compared to disability services, children identified in hospital or ambulatory data alone were more likely to be female, older at first contact, and to live in major cities and less disadvantaged areas (Supplementary Table S3).

Age and sex-specific estimates of the prevalence of ASD in NSW are presented in Table 2.

**Table 2:** Age and sex-specific prevalence of autism spectrum disorder by data source, New South Wales, Australia

Age (years)	Cases				Population	Prevalence			
	Disability Services	Hospital Admissions	Ambulatory Mental Health	Total		Disability Services	Hospital Admissions	Ambulatory Mental Health	Total
Total									
3	6579	678	46	6901	1,161,758	0.6%	0.1%	0.0%	0.6%
6	8543	1593	238	9285	870,809	1.0%	0.2%	0.0%	1.1%
9	6332	1584	480	7192	583,518	1.1%	0.3%	0.1%	1.2%
12	3273	1048	503	3930	301,940	1.1%	0.4%	0.2%	1.3%
Male Children									
3	5201	523	35	5442	597,364	0.9%	0.1%	0.0%	0.9%
6	6798	1239	188	7346	447,357	1.5%	0.3%	0.0%	1.6%
9	5043	1252	375	5693	300,155	1.7%	0.4%	0.1%	1.9%
12	2638	837	393	3150	155,494	1.7%	0.5%	0.3%	2.0%
Female Children									
3	1378	155	11	1459	564,394	0.2%	0.0%	0.0%	0.3%
6	1745	354	50	1939	423,452	0.4%	0.1%	0.0%	0.5%
9	1289	332	105	1499	283,363	0.5%	0.1%	0.0%	0.5%
12	635	211	110	780	146,446	0.4%	0.1%	0.1%	0.5%

**Fig. 1:**
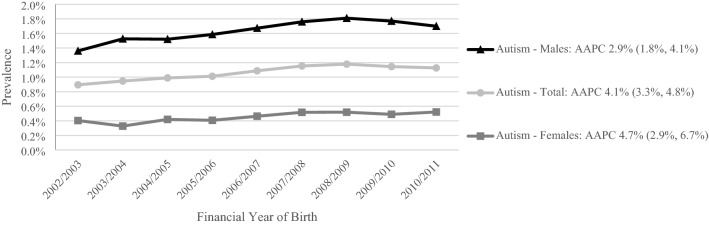
Sex-specific prevalence of autism spectrum disorder at age 6 by individual financial year of birth in New South Wales, Australia. AAPC = Average Annual Percent Change. AAPC calculated using negative binomial regression to account for overdispersion

The overall prevalence of ASD was 0.6% at age 3, 1.1% at age 6, 1.2% at age 9, and 1.3% at age 12 years, and was 3–4 times higher among male children at all ages (1.6% of males vs. 0.5% of females at age 6). Cases identified from disability services data were the largest component of all prevalence estimates. The prevalence of ASD at age 6 years increased an average of 4.1% (95% Confidence Interval (CI), 3.3%, 4.8%) per year from 0.9% of children born in 2002/2003 to 1.1% of children born in 2010/2011. The average annual increase was higher for females than males, 4.7% (95% CI, 2.9%, 6.7%) vs. 2.9% (95% CI, 1.8%, 4.1%) (Fig. 1).

**Fig. 2:**
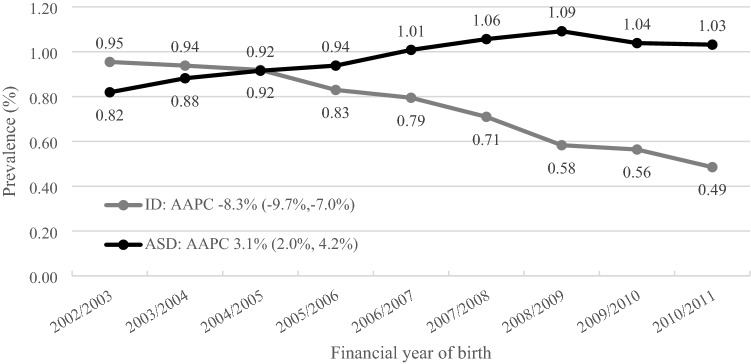
Prevalence of intellectual disability and autism spectrum disorder by age 6 identified in disability services data in New South Wales, Australia. AAPC = Average annual percent change, ASD = Autism spectrum disorder, ID = Intellectual disability. Intellectual disability and autism status determined by primary and secondary disability groups documented in disability services data. AAPC calculated using negative binomial regression to account for overdispersion

Looking at disability services data alone, the prevalence of ASD by age 6 years increased an average of 3.1% per year (95% CI, 2.0%, 4.2%) from 0.8% of children born in 2002/2003 to 1.0% of children born in 2010/2011 (Fig. 2).

In contrast, the prevalence of ID at age 6 years identified in disability services data decreased an average of 8.3% per year (95% CI, 7.0%, 9.7%) from 1.0% of children born in 2002/2003 to 0.5% of children born 2010/2011. The proportion of children identified with both autism and intellectual disability in disability services data decreased from 50.0% of ASD cases to 15.2% of ASD cases over the study period.

## Discussion

Using three routinely collected data sources in NSW, most cases of ASD were ascertained from disability services data, with children more likely to be male, younger at first contact, live outside major cities and from more disadvantaged areas compared with cases identified in hospital or ambulatory data alone. Based on these data sources, we estimated the prevalence of ASD to be 1.1% by age 6 and 1.3% by 12 years of age. Overall, ASD prevalence among 6-year-old children increased an average of 4.1% per year over the study period. Prevalence rates were 3–4 times higher among male children but increased faster among female children.

The estimated 1.3% ASD prevalence from this study is higher than the most recent published population-level estimate from Western Australia, 0.5% of children born 1993–2005 (Bourke et al., 2016). That estimate was based on data from the Intellectual Disability Exploring Answers (IDEA) database, which identifies cases of intellectual and developmental disabilities from receipt of disability services and educational supports in schools (Petterson et al., 2005). The estimate from the current study is consistent with a more recent study of data on new autism treatment plans from Medicare, Australia’s universal healthcare program, which reported a cumulative incidence of 1.1% in 4-year-olds (May & Williams, 2018). Medicare reimbursement for autism treatment plans covers those with an autism diagnosis as well as those diagnosed with other pervasive developmental disorders. However, the prevalence estimate from the current study is lower than the most recent Australian estimates based on parental surveys, including an ongoing cohort study in Victoria (2.6–4.4%) (May et al., 2020) and the national Survey of Disability, Ageing and Carers (2.8%) (Australian Bureau of Statistics, 2019). It is also lower than the 2.3% prevalence among 8-year-old children in 2018 reported by the ADDM network, the main active autism surveillance program in the US (Maenner et al., 2021).

Compared to all births in NSW, children identified with ASD were more likely to live in regional and more disadvantaged areas. A previous study in Western Australia found ASD was associated with higher socioeconomic status (Leonard et al., 2011), but the association between socioeconomic disadvantage and ASD is complicated with cohort studies in different populations reporting contradictory results (Delobel-Ayoub et al., 2015; Durkin et al., 2017; Kelly et al., 2019; Roman-Urrestarazu et al., 2021; Yu et al., 2021). In the current study, disability services data only captured individuals who were receiving publicly funded services. A 2016 survey of Australian healthcare providers reported substantial wait times for autism diagnostic assessments, particularly in the public sector, which had a median wait of 16 weeks from time of referral up to a maximum of two years (Taylor et al., 2016). A study of referral and assessment pathways at a tertiary hospital in Melbourne reported children waited an average of 9 months for an ASD assessment (Bernie et al., 2021). Individuals with more resources may choose to avoid the public system and receive services from private providers (Kayrouz et al., 2014). In addition, regional differences in prevalence estimates may be shaped by differences in access to and availability of services.

As expected, we found that ASD prevalence increased over the study period, however the 4.1% average annual increase is slower than previous studies in Australia (May et al., 2020; Nassar et al., 2009) and the United States (Nevison & Blaxill, 2017; Zablotsky et al., 2019; Baio et al., 2018; Maenner et al., 2021). For example, a recent study of two Australian birth cohorts born four years apart (2000 vs. 2004), reported a 69% increase in prevalence of ASD from 2.6 to 4.4% at age 12–13 (May et al., 2020). The authors suggest that the dramatic increase was likely caused by changes in diagnostic criteria and the introduction of new funding from the Helping Children with Autism (HCWA) program, for which only their younger cohort was eligible. In July 2008, the Australian government introduced the HCWA program, which provided funding for early intervention services to registered children with an ASD diagnosis aged six years or younger (Bent et al., 2015). To receive these resources, an ASD diagnosis may have been emphasized rather than other neurodevelopmental conditions or intellectual disability (Croen et al., 2002; Nassar et al., 2009; Shattuck, 2006). In contrast, we found the prevalence of ID in disability services data decreased substantially over the course of the study period. This was unexpected and this decrease has not been reported in prior studies from Western Australia (Bourke et al., 2016; Nassar et al., 2009). This decrease is likely due in part to structural changes in funding for disability services in Australia (May et al., 2018; Australian Institute of Health and Welfare, 2018).

Comparing the three data sources used in this study, there was only limited overlap in individuals identified with three-quarters of cases found in disability services data alone. Limited identification from hospital admission data is expected, given that, while children with ASD are more likely to have comorbidities and to be admitted to hospital in early childhood (Alexeeff et al., 2017; Atladóttir et al., 2010; Brooks et al., 2021), the inpatient environment is not a primary site of treatment for neurodevelopmental disorders. A previous linked data study in New South Wales reported only 16% of individuals identified with intellectual disability were identified in hospital admission data alone, with the rest identified in disability services data (Reppermund et al., 2017). Similarly, a study in Western Australia found that hospital admission data poorly identified individuals with intellectual disability when compared to disability services and education data (Bourke et al., 2018).

Limited identification from ambulatory mental health data is more surprising, given the high level of mental health comorbidities experienced by individuals with autism. However, there are multiple barriers for individuals with ASD to receive mental health services, including discomfort and limited knowledge about ASD among some mental health practitioners (Adams & Young, 2021). In addition, the dataset used in this study included only one diagnosis code per encounter and did not include visits with general practitioners and private specialists. A validation study in Canada reported low sensitivity (17%) when identifying ASD from mental health outpatient data, with most cases identified in physician billing data (Dodds et al., 2009). Based on these results, hospital admission data and ambulatory mental health contacts alone do not sufficiently capture cases of ASD in New South Wales. However, it is important to link the three sources together because the 13% of children identified outside of disability services may represent a distinct population. Older at first contact, these children may have less severe autism symptoms or may lack access to services. Future studies should investigate this population to identify opportunities for improving access to autism services.

This study has several strengths including use of population-level data from three different service types - inpatient, outpatient, and community services. To our knowledge, this is the first study to estimate ASD prevalence in NSW using data linkage, and, while not complete in their coverage, these datasets together likely represent the most comprehensive data currently available in NSW. Under-enumeration may be possible, either because ASD cases were never diagnosed, or because they only received services from other sources, such as private providers and school accommodations. This lack of a “gold standard” prevented calculation of sensitivity and specificity metrics for each data source.

Previous validation studies in Australia, (Bourke et al., 2018) North America, (Burke et al., 2014; Dodds et al., 2009) and the Nordic countries (Lampi et al., 2010; Lauritsen et al., 2010) found that diagnosis codes for ASD in hospital admission and ambulatory mental health records produce few false positives. This was true when using medical record abstraction (Burke et al., 2014; Lauritsen et al., 2010), diagnostic interviews (Dodds et al., 2009; Lampi et al., 2010), or registries (Bourke et al., 2018) as a gold standard. As such, cases identified in hospital admission and ambulatory mental health data in NSW are expected to be true cases. Identifying cases from health and disability data sources can also reduce bias and over-reporting that may result from parental reporting.

Based on three routinely collected data sources, 1.3% of children born in NSW developed ASD by age 12. Identifying cases of ASD is important for evaluating prevalence and the health and wellbeing of children in a population. In this study we found disability services data were the best single source for identifying children with ASD in New South Wales, while hospital admissions or ambulatory mental health data alone should not be used for this purpose. However, if possible, all three data sources should be linked together to ensure children diagnosed at an older age and children without access to services are included.

### Supplementary Information

Below is the link to the electronic supplementary material.

Supplementary material 1 (DOCX 23.0 kb)
