# Supplementary material for: Estimating the Prevalence of Autism Spectrum Disorder in New South Wales, Australia: A Data Linkage Study of Three Routinely Collected Datasets
Source: J Autism Dev Disord. 2023 Jan 18;54(4):1558–66. doi: 10.1007/s10803-022-05887-3 (PMC10981615; doi:10.1007/s10803-022-05887-3)
Supplement: Supplementary file 1 — Supplementary material 1 (DOCX 23.0 kb) [file 10803_2022_5887_MOESM1_ESM.docx]

**Supplementary Table S1.** Disability groups included in Disability Services National Minimum Dataset.*

| **Disability Group** | **Description** |
| --- | --- |
| 1. Intellectual | Conditions appearing in the developmental period (age 0–18 years) associated with impairments of mental functions, difficulties in learning and performing certain daily life skills and limitations of adaptive skills in the context of community environments compared to others of the same age. |
| 2. Specific learning/Attention Deficit Disorder (ADD) | A group of disabilities, presumed due to central nervous system dysfunction rather than an intellectual disability, covering significant difficulties in the acquisition and use of organisational skills, listening, speaking, reading, writing, reasoning or mathematical skills. |
| 3. Autism (including Asperger’s syndrome and Pervasive Developmental Delay) | Pervasive developmental disorders involving disturbances in cognition, interpersonal communication, social interactions and behaviour (in particular obsessive, ritualistic, stereotyped and rigid behaviours). |
| 4. Physical | Conditions that are attributable to a physical cause or impact on the ability to perform physical activities, such as mobility. Includes impairments of the neuromusculoskeletal systems. |
| 5. Acquired brain injury | Disabilities arising from damage to the brain acquired after birth. May be a result of accidents, stroke, brain tumours, infection, poisoning, lack of oxygen or degenerative neurological disease. |
| 6. Neurological (including epilepsy and Alzheimer’s disease) | Impairments of the nervous system occurring after birth, includes epilepsy and organic dementias (for example, Alzheimer’s disease) as well as such conditions as multiple sclerosis and Parkinson’s disease |
| 7. Deafblind (dual sensory) | Dual sensory impairments associated with severe restrictions in communication, and participation in community life. Deafblindness is not just vision impairment with a hearing loss, or a hearing loss with a vision impairment. |
| 8. Vision | Blindness and vision impairment (not corrected by glasses or contact lenses), which can cause severe restriction in communication and mobility, and in the ability to participate in community life. |
| 9. Hearing | Encompasses deafness, hearing impairment, hearing loss |
| 10. Speech | Encompasses speech loss, impairment and/or difficulty in being understood. |
| 11. Psychiatric | Recognisable symptoms and behaviour patterns, frequently associated with distress, which may impair personal functioning in normal social activity. Includes schizophrenia, affective disorders, anxiety disorders, addictive behaviours, personality disorders, stress, psychosis, depression and adjustment disorders. |
| 12. Developmental delay | Applies to children aged 0–5 where conditions have appeared in the early developmental period, but no specific diagnosis has been made and the specific disability group is not yet known. |

**Descriptions from the Disability Services National Minimum Data Set* (Australian Institute of Health and Welfare, 2016).

**Supplementary Table S2:** Overlap in identification of autism spectrum disorder between three routinely collected data sources in New South Wales, Australia.

|  |  | ASD Cases | | |
| --- | --- | --- | --- | --- |
|  |  | Total | Male | Female |
| Data source | Comparison | n (%) | n (%) | n (%) |
| DS | Total | 11,206 (100.0) | 8879 (100.0) | 2327 (100.0) |
|  | HOSP | 1708 (15.2) | 1377 (15.5) | 331 (14.2) |
|  | AMH | 386 (3.4) | 314 (3.5) | 72 (3.1) |
| HOSP | Total | 2912 (100.0) | 2270 (100.0) | 642 (100.0) |
|  | DS | 1708 (58.7) | 1377 (60.7) | 331 (51.6) |
|  | AMH | 220 (7.6) | 168 (7.4) | 52 (8.1) |
| AMH | Total | 971 (100.0) | 760 (100.0) | 211 (100.0) |
|  | DS | 386 (39.8) | 314 (41.3) | 72 (34.1) |
|  | HOSP | 220 (22.7) | 168 (22.1) | 52 (24.6) |

*AMH = Ambulatory mental health encounters, ASD = Autism spectrum disorder, DS = Disability services, HOSP = Hospital admissions. Individuals missing information on sex are excluded from this table.*

**Supplementary Table S3:** Comparing characteristics of children with autism spectrum disorder identified in disability services data vs. other sources in New South Wales, Australia, born 2002-2015.

|  | Total Population | Autism Spectrum Disorder | | P value^a^ |
| --- | --- | --- | --- | --- |
|  |  | (n=12,921) | |  |
|  |  | Disability Services | Not in Disability services |  |
|  | (n=1,211,834) | (n=11,210) | (n=1711) |  |
| Characteristics | n (%) | n (%) | n (%) |  |
| Child sex |  |  |  | <0.001 |
| Male | 622,949 (51.4) | 8879 (79.2) | 1281 (74.9) |  |
| Female | 588,374 (48.6) | 2327 (20.8) | 429 (25.1) |  |
| Age at first contact (years) |  |  |  | <0.001 |
| 0-2 |  | 3545 (31.6) | 123 (7.2) |  |
| 3-5 |  | 5988 (53.4) | 538 (31.4) |  |
| 6-8 |  | 1085 (9.7) | 531 (31.0) |  |
| 9-11 |  | 467 (4.2) | 324 (18.9) |  |
| 12-15 |  | 125 (1.1) | 195 (11.4) |  |
| Residence at birth |  |  |  | <0.001 |
| Major city | 936,467 (77.3) | 7849 (70.0) | 1319 (77.1) |  |
| Inner regional | 199,360 (16.5) | 2701 (24.1) | 287 (16.8) |  |
| Outer regional | 54,080 (4.5) | 569 (5.1) | 93 (5.4) |  |
| Remote/Very remote | 7067 (0.6) | 52 (0.5) | 8 (0.5) |  |
| Missing | 14,860 (1.2) | 39 (0.4) | 4 (0.2) |  |
| Socioeconomic disadvantage at birth |  |  |  | <0.001 |
| Quintile 1 – Most disadvantaged | 264,570 (21.8) | 2826 (25.2) | 432 (25.3) |  |
| Quintile 2 | 285,574 (23.6) | 3338 (29.8) | 456 (26.7) |  |
| Quintile 3 | 230,767 (19.0) | 2247 (20.0) | 300 (17.5) |  |
| Quintile 4 | 186,186 (15.4) | 1475 (13.2) | 274 (16.0) |  |
| Quintile 5 – Least disadvantaged | 229,877 (19.0) | 1285 (11.5) | 245 (14.3) |  |
| Missing | 14,860 (1.2) | 39 (0.4) | 4 (0.2) |  |

*^a^ Pearson’s Chi-Squared test comparing characteristics of children identified in disability services and children identified in other sources.*
